# Co-Occurrence of Germline Genomic Variants and Copy Number Variations in Hereditary Breast and Colorectal Cancer Patients

**DOI:** 10.3390/genes14081580

**Published:** 2023-08-03

**Authors:** Luiza Côrtes, Tatiane Ramos Basso, Rolando André Rios Villacis, Jeferson dos Santos Souza, Mads Malik Aagaard Jørgensen, Maria Isabel Achatz, Silvia Regina Rogatto

**Affiliations:** 1 Department of Clinical Genetics, University Hospital of Southern Denmark, Beriderbakken 4, 7100 Vejle, Denmark; luiza.cortes@unesp.br (L.C.); tatianebasso2015@gmail.com (T.R.B.); mads.jorgensen@rsyd.dk (M.M.A.J.); 2 Tocogynecoly Graduation Program, Botucatu Medical School, University of São Paulo State—UNESP, Botucatu 18618-687, SP, Brazil; 3 Department of Genetics and Morphology, Institute of Biological Sciences, University of Brasília—UnB, Brasília 70910-900, DF, Brazil; rolando.andre@unb.br; 4 Health Technology Institute, SENAI CIMATEC, Salvador 41650-010, BA, Brazil; jeferson169087@gmail.com; 5 Cancer Genetics Unit, Oncology Branch, Hospital Sirio-Libanês, São Paulo 01308-050, SP, Brazil; miachatz@mochsl.org.br; 6 Institute of Regional Health Research, Faculty of Health Sciences, University of Southern Denmark, 5000 Odense, Denmark; 7 Danish Colorectal Cancer Center South, 7100 Vejle, Denmark

**Keywords:** hereditary breast and ovarian cancer syndrome, colorectal cancer, whole exome sequencing, *PMS2*, copy number alteration

## Abstract

Hereditary Breast and Ovarian Cancer (HBOC) syndrome is an autosomal dominant disease associated with a high risk of developing breast, ovarian, and other malignancies. Lynch syndrome is caused by mutations in mismatch repair genes predisposing to colorectal and endometrial cancers, among others. A rare phenotype overlapping hereditary colorectal and breast cancer syndromes is poorly characterized. Three breast and colorectal cancer unrelated patients fulfilling clinical criteria for HBOC were tested by whole exome sequencing. A family history of colorectal cancer was reported in two patients (cases 2 and 3). Several variants and copy number variations were identified, which potentially contribute to the cancer risk or prognosis. All patients presented copy number imbalances encompassing *PMS2* (two deletions and one duplication), a known gene involved in the DNA mismatch repair pathway. Two patients showed gains covering the *POLE2* (cases 1 and 3), which is associated with DNA replication. Germline potentially damaging variants were found in *PTCH1* (patient 3), *MAT1A*, and *WRN* (patient 2). Overall, concurrent genomic alterations were described that may increase the risk of cancer appearance in HBOC patients with breast and colorectal cancers.

## 1. Background

Breast cancer (BC) and colorectal cancer (CRC) are frequent neoplasms, with more than 4 million people worldwide diagnosed yearly [1]. Individuals with hereditary cancer syndromes are at an increased risk of developing second primary tumors, such as BC and CRC [2,3].

Pathogenic variants in known cancer-predisposition genes are responsible for 5% to 10% of BC and CRC cases [4]. The most common pathogenic variants related to hereditary breast and ovarian cancer syndrome (HBOC) occur in *BRCA1* and *BRCA2* genes [5]. Lynch syndrome (LS) is a common condition of hereditary colorectal cancer syndromes. LS is predominantly associated with CRC and endometrial cancer and an increased lifetime risk of developing multiple primary cancer types (small bowel, urothelium, biliary tract, and stomach). Although under debate, several studies suggested that BC may be included as extracolonic cancer in LS [6,7]. LS is related to pathogenic germline variants in DNA mismatch repair (MMR) genes (*MLH1*, *MSH2*, *MSH6*, *PMS2*) or *EPCAM* and [8,9]. Of note, many patients that fulfill the criteria of hereditary cancer predisposition syndromes do not exhibit pathogenic variants in the main genes described as altered in these syndromes [4,10,11].

In addition to germline genetic variants, copy number variations (CNVs) can explain the heritability of several cancer syndromes, including HBOC and LS [12,13,14,15]. Previously, we reported a germline deletion mapped in the transcriptional regulatory region of *ROBO1* in three patients fulfilling the criteria of hereditary breast and colorectal cancer [13]. We also described rare CNVs in 11 of 22 patients with multiple primary tumors, including the *EPCAM/MSH2* deletion in one Lynch syndrome patient [14]. Rare germline CNVs have been reported in patients with a family history of BC, negative for *BRCA1* and *BRCA2* pathogenic variants [13,16,17].

Multiple primary cancers occur in a substantial proportion of cancer patients (10–25%) [18,19]. Patients with breast cancer are at higher risk of developing CRC, and patients with CRC show an increased incidence of BC [3,20,21]. Pathogenic variants of *BRCA1, BRCA2, MLH1, MSH2, MSH6, PALB2, CHEK2, ATM,* and *TP53* were associated with an increased risk of breast and colorectal cancer [4,22,23]. Herein, the whole exome sequencing results were described in three unrelated patients with a personal and/or family history of BC and CRC. These patients have no germline pathogenic variants in the major predisposition genes related to their clinical phenotype.

## 2. Methods

### 2.1. Patients

Three BC patients with second primary CRC were selected from a cohort of 445 patients that fulfilled the testing criteria for inherited breast and ovarian cancer (National Comprehensive Cancer Network—NCCN—Guidelines Genetic/Familial High-Risk Assessment: Breast, Ovarian, and Pancreatic v2.2021). Patients prospectively consented to blood sample sequencing via whole exome sequencing. The study was conducted according to the ethical guidelines and regulations from the Declaration of Helsinki, and the Human Research Ethics Committee approved the study (CONEP Protocol # 2136/15). The patients were treated at A.C. Camargo Cancer Center, São Paulo, Brazil. All patients were tested for *TP53*, *BRCA1*, *BRCA*, *MLH1*, and *MSH2* by Sanger sequencing, and no pathogenic variants were found.

### 2.2. DNA Extraction, Whole Exome Sequencing (WES), and Data Analysis

Genomic DNA from peripheral blood lymphocytes was extracted using the QIAamp DNA Blood Mini QIAcube Kit (Qiagen, Hilden, Germany), following the manufacturer’s instructions. DNA quality and quantification assessments were performed with the Genomic DNA ScreenTape assay (Agilent Technologies Inc., Santa Clara, CA, USA) and the Qubit dsDNA BR assay Kit (Thermo Fisher Scientific Inc., Waltham, MA, USA). Libraries were prepared using TruSeq Rapid (Illumina, San Diego, CA, USA), and the pair end (PE150) sequencing was performed on a NextSeq550 System (Illumina, San Diego, CA, USA), following the manufacturer’s recommendations. The sequence reads were aligned (Burrows-Wheeler Alignment, BWA-mem) to the human genome using the UCSC hg19, recalibrated, and realigned with the Genome Analysis Toolkit (GATK) [24]. The alignment statistics were performed with Sequence Alignment/Map (SAMtools), Picard, and Binary Alignment Map (BAMtools). Variant calling and merging were carried out with GATK HaplotypeCaller.

Germline variants annotation and selection were carried out using VarSeq™ version 2.2.2 (Golden Helix Inc., Bozeman, MT, USA). Single nucleotide variants (SNVs) and InDels were identified and classified using ACMG guidelines (revised in November 2022). Variants mapped in homopolymer regions or predicted as benign (B) or likely benign (LB) (ACMG or ClinVar classification), and those with frequency > 0.2 in the GnomAD v2.1 (The Genome Aggregation Database) and-/or in the ABRaOM (Online Archive of Brazilian Mutations) datasets were excluded [25,26]. The variants were manually curated using the Genome Browse software (Golden Helix Inc.). Only nonsynonymous, protein-damaging, and rare variants were included and manually curated. Pathogenic (P) and likely pathogenic (LP) variants were selected using ACMG and ClinVar classification. The variants of uncertain significance (VUS) potentially damaging were selected using deleterious effects filtering. Variants associated with loss of function or splice altering predictions (Ada scores > 0.6) or predicted as damaging in at least two independent functional prediction algorithms, including Sorting Intolerant From Tolerant (SIFT) [27], Polymorphism Phenotyping v2—Polyphen2 HVAR [28], MutationTaster [29], MutationAssessor [30], Functional Analysis through Hidden Markov Models—FATHMM, and FATHMM-MKL Coding [31], or Combined Annotation Dependent Depletion (CADD) score > 3 were selected. The list of variants was compared with the Catalogue Of Somatic Mutations In Cancer (COSMIC, https://cancer.sanger.ac.uk/cosmic, accessed on 30 May 2023).

Genomic CNVs were retrieved using CNV caller on target regions algorithm (Golden Helix VarSeq v.2.2.2). CNVs with a *p*-value < 0.001 were included, and those variants with a frequency ≥ 0.01 on the Database of Genomic Variant (DGV) were excluded. A circus plot was constructed to visualize the CNVs identified [32].

## 3. Results

**Figure 1:**
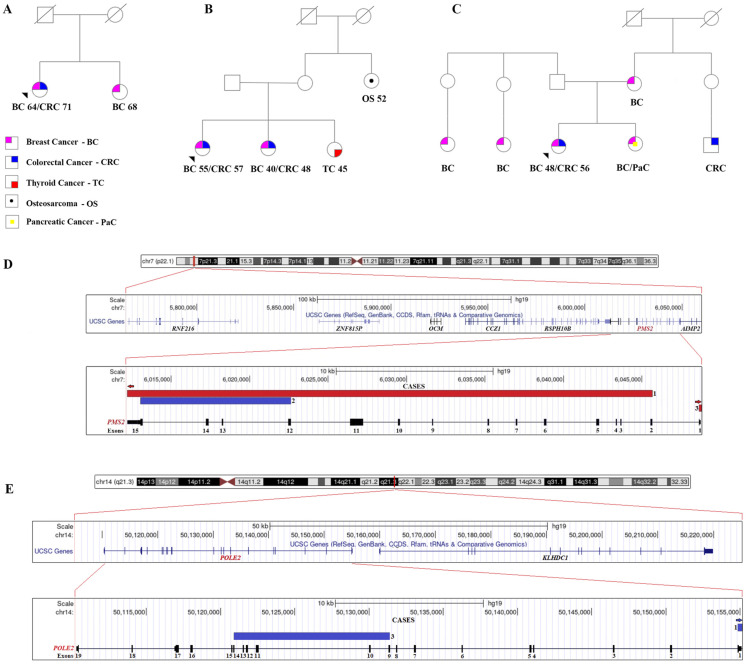
Representative pedigree of three index patients following the hereditary breast and ovarian cancer syndrome criteria. Index patients 1 (**A**), 2 (**B**), and 3 (**C**). The arrowheads indicate the index patients. The numbers below the symbols represent the age at diagnosis of each tumor type. (**D**) Graphical representation of *PMS2* gene. *PMS2* gene spans 15 exons and 862 amino acids in length. Exons 2 to 15 deletion of the *PMS2* gene is represented in red (Case 1). In blue, gains of exons 12 to 15 are detected in Case 2. Patient 3 presented the exon 1 loss of *PMS2.* (**E**). *POLE2* gains were detected in index patients 1 and 3.

The most relevant clinical and pathological features of each patient are summarized in Table 1. The patients met the NCCN v2.2021 criteria for HBOC and Hereditary Breast and Colorectal Cancer (HBCC), according to Meijers-Heijboer et al. [33] and Naseem et al. [22]. The representative pedigrees of these patients are depicted in Figure 1. Index patient 1 developed bilateral BC (64 years) and CRC (at age 71); her sister had BC diagnosed at age 68 (Figure 1A). Patient 2 was diagnosed with synchronous bilateral BC (55 years) and CRC (57 years). One sister had BC (40 years) and CRC (48 years), thyroid cancer was reported in another sister (45 years), and her maternal aunt presented osteosarcoma at age 52 (Figure 1B). Patient 3 was diagnosed with BC (48 years) and CRC (56 years), had four relatives with BC (mother, sister, and two paternal cousins), and a maternal cousin with CRC (Figure 1C).

**Figure 2:**
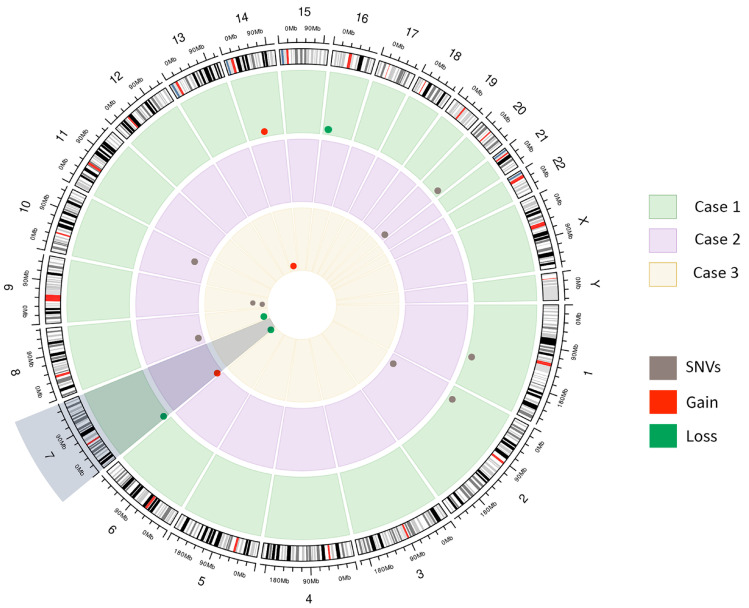
Circus plot showing selected germline genomic variants (SNVs and CNVs) detected in three index patients. *PMS2* copy number variations are highlighted in grey.

Germline SNVs and CNVs were investigated in all three index patients (Figure 2, Table 2 and Table S1). Patient 1 presented the germline LP variant *CYP1B1* c.1159G > A (Table 2), along with 49 VUS, including two missense variants *NTRK1* c.16C > T and *NFATC2* gene c.1952G > A reported as cancer-associated (COSMIC, https://cancer.sanger.ac.uk/cosmic (accessed on 30 May 2023)). This patient also presented 212 rare CNVs (*p* < 0.001), such as the heterozygous deletion of the *PMS2* gene (start: 5764955, end: 6045662, spanning 280,707 base pairs, and affecting exons 2 to 15) (Figure 1D), a gain of *POLE2* gene (start: 50154854, end: 50160008) (Figure 1E), and a heterozygous deletion of *NOMO1* (start: 14946287, end: 14959012) (Table 2). Copy number variants of *PMS2* were also found in all index patients, while gains of *POLE2* were detected in patients 1 and 3.

The index patient 2 presented three LP variants (*SLC3A1* c.1400T > C, *MAT1A* c.826dupG, and *PTGIS* c.824G > A) and 59 VUS, including the missense variant *WRN* c.95A > G, which is also in the COSMIC gene list. Seventeen rare CNVs were identified, including a gain of 9592 bp in the *PMS2* gene (start: 6013030, end: 6022622, spanning exons 12 to 15) (Figure 1D and Table 2). This region has high homology with the *PMS2CL* pseudogene.

The index patient 3 presented 33 VUS, including the missense variants *GNAQ* c.286A > T and *PTCH1* c.140G > T, previously described as cancer-associated genes (COSMIC gene list). Among the 150 rare CNVs found in this patient, a heterozygous deletion of *PMS2* (start: 6048628, end: 6049129, 501 bp at exon 1) was identified (Figure 1D), a region with no homology with the *PMS2CL* pseudogene. Additionally, a heterozygous deletion of the *ASH2L* gene (start: 37914599, end: 37963256) and a gain in *POLE2* (start: 50120873, end: 50131392) (Figure 1E) were found (Table 2).

## 4. Discussion

The accumulation of germline variants and CNVs in cancer-related genes may explain the development of multiple primary tumors in individual patients [34]. In this study, we investigated the germline whole exome sequencing of three breast and colorectal cancer individuals fulfilling the HBOC and HBCC criteria. Pathogenic and likely pathogenic variants were not detected in two index patients. All patients presented variants with uncertain significance; a set of these genes was described as cancer-related. Moreover, SNVs and CNVs that may contribute to the cancer risk were identified in all three index patients. Previously, we reported that early-onset rectal cancer (<40 years) patients exhibited a significantly higher number of germline variants, as well as multiple variants in oncogenes, tumor suppressors, and mismatch repair genes associated with hereditary colorectal cancer [35]. In early-onset breast cancer, concurrent germline variants of *BRCA1*, *BRCA2*, *PMS2*, and *PALB2* were reported [36]. Analysis of hereditary cancer cases (732 BC patients, 189 CRC patients, and 490 cancer-free elderly controls) revealed that 1% presented concurrent germline pathogenic variants in *BRCA1*, *BRCA2*, *MUTYH*, *ATM*, *CHECK2*, *NBN*, and *RAD50* [37]. The germline genomic profile of BC/CRC patients fulfilling the criteria of HBOC and HBCC is poorly explored in the literature.

Patient 1 presented the LP variant *CYP1B1* c.1159G > A. The protein encoded by *CYP1B1* plays a role in the metabolism of steroid hormones and the progression of certain cancers, such as breast and prostate cancer [38,39]. Previous single nucleotide polymorphism studies of *CYP1B1* showed an association with an increased risk of developing BC and CRC [40,41]. In CRC samples, high *CYP1B1* expression was significantly associated with lower overall and disease-free survival rates [42]. Among the 49 VUS identified in the index patient 1, two genes were cancer-related: *NTRK1*c.16C > T and *NFATC2* c.1952G > A. Gene fusions involving *NTRK1* can confer oncogenic potential in several tumors, including BC and CRC [43,44]. These genetic fusions in the tyrosine receptor kinases have emerged as targets for cancer therapy [45]. *NFATC2* codifies a member of transcription factors activated by calcium signaling pathways that acts in the immune response control [46,47]. *NFATC2* regulates the self-renewal capacity and tumorigenic initiation of tumor stem cells and is a promising therapeutic target for colorectal cancer treatment [48]. Overall, *CYP1B1, NTRK1,* and *NFATC2* are not related to cancer risk but have potential roles as prognostic markers and actionable drug targets.

Index patient 1 presented 213 rare copy number variations (*p* < 0.0001), including those mapped in *PMS2*, *POLE2*, and *NOMO1* genes. A heterozygous deletion of the *PMS2* gene was also found in patient 3, while a significant gain was detected in patient 2. A heterozygous pathogenic deletion of *PMS2* (exons 13–15) was reported in two unrelated BC patients with no clinical or familial criteria for LS phenotype [15]. Interestingly, this *PMS2* pathogenic deletion was also identified in two patients with transverse colon cancer [49]. The *PMS2* gene (PMS1 homolog 2, mismatch repair system component, mapped on 7p22.1) encodes a key protein involved in the DNA mismatch repair, and it is associated with LS [50]. The molecular diagnosis of *PMS2* in hereditary cancer syndromes is challenging due to a family of pseudogenes highly homologous to PMS2 [51,52]. Fourteen pseudogenes showed high homology with the 5′end (exons 1 to 5) of *PMS2*, while the pseudogene *PMS2CL* shares high sequence similarity to exons 9 and exons 11–15 of *PMS2* [51,52]. The large heterozygous deletion of *PMS2* (280,707 bp; spanning exons 2 to 15) found in patient 1 has partial homology with the pseudogenes and, therefore, can impact the cancer risk of this patient and her family members. The *PMS2* gain identified in patient 2 and the heterozygous loss detected in patient 3 are questionable as to their clinical impact. The biological significance of these alterations for cancer risk should be confirmed. However, protocols to ensure CNVs have challenges: they are expensive, time-consuming, and show gene-specific limitations, such as the availability of MLPA kits (MRC Holland), and do not always result in practical conclusions [53,54]. Although some alterations should be better investigated, reports presenting findings in rare diseases are critical to identifying their clinical and biological consequences [55]. A study focusing on CRC at a young age showed that women with *PMS2* germline variants had a 3-fold increased risk for BC development [56]. It is reasonable to suggest that CNVs of *PMS2* contribute to the cancer risk in HBOC and HBCC patients. Copy number gains of *POLE2* were identified in two patients (1 and 3). The POLE2 protein (centromere-associated protein E) is a subunit of DNA polymerase epsilon (Pol ε), which is one of the main enzymes involved in DNA replication [57]. *POLE2* mutations have been associated with colorectal cancer, but the *POLE2* CNV related to hereditary cancer syndrome is a new finding [57,58,59]. Gains of *POLE2* can alter the protein function and contribute to cancer risk. Heterozygous loss of *NOMO1* (start: 14946287, end: 14959012) was also detected in index case 1. Colorectal cancer frequently exhibits *NOMO1* deletion [60,61]. This biomarker is a potential therapeutic target for early-onset colorectal cancer [60].

Index patient 2 presented three likely pathogenic variants *SLC3A1* c.1400T > C, *MAT1A* c.826dupG, and *PTGIS* c.824G > A. Breast cancer cells have elevated expression of *SLC3A1,* which accelerates cysteine uptake and accumulates reduced glutathione and decreases reactive oxygen species in tumor cells [62,63]. Dysregulation of *MAT1A* in tumor cells was associated with replication errors and genetic instability [64,65]. Furthermore, *MAT1A* dysregulation increases homocysteine levels, promoting cancer progression [66]. The *PTGIS* (Prostaglandin I2 Synthase) gene encodes a cytochrome P450 oxidase family member. In colorectal cancer, *PTGIS* dysregulation may contribute to chromosomal instability and tumor progression [67]. High *PTGIS* expression can promote infiltration of immune cells, such as tumor-associated macrophages and regulatory T cells, into the tumor microenvironment, which impairs the prognosis of patients with lung, ovarian, and gastric cancer [68,69]. The missense variant *WRN*c.95A > G was also identified among the 59 VUS of index case 2. The tumor suppressor *WRN* encodes an enzyme from the RECQ gene family, which is essential for maintaining genomic stability [70]. Germline variants of *WRN* were described in HBOC and early-onset familial colorectal cancer [71,72,73]. The heterozygous loss at exon 1 of *PMS2* was identified among the 17 rare CNVs detected in this patient, as discussed above. We suggest that *MAT1A* and *WRN* variants and *PMS2* loss could contribute to the hereditary cancer predisposition phenotype observed in this family.

Although index patient 3 had no P/LP variants, 33 VUS, and two potentially damaging missense variants, *GNAQ*c.286A > T and *PTCH1*c.140G > T (COSMIC) were identified. Mutations in the *GNAQ*/*GNA11* gene activate the MAP kinase signaling pathway, a key factor in the development of uveal melanoma [74]. The *GNAQ* c.286A > T variant detected in the index patient was reported in a small percentage of colorectal cancers assessed by gene panel sequencing [75]. Germline inactivating mutations in *PTCH1* were associated with Gorlin Syndrome, a genetic predisposition to multiple basal cell carcinoma and medulloblastoma [76]. Recently, Yoshida et al. reported a *PTCH1* germline variant in one patient with second malignant neoplasms (primary: medulloblastoma; second: osteosarcoma and acute myeloid leukemia) [77]. Germline pathogenic *PTCH1* variant was also described in one colorectal cancer patient whose cancer showed microsatellite instability and wild-type MMR genes [78]. Germline variants in cancer predisposition genes not related to the patient phenotype need investigation to result in adequate clinical management.

Moreover, 150 rare CNVs were found in the index patient 3. Among them, a heterozygous deletion of 501 bp in *PMS2* (exon 1), as described above, a heterozygous deletion of *ASH2L*, and a gain of *POLE2* (start: 50120873, end: 50131392). *ASH2L* gene encodes a protein involved in the epigenetic regulation and transcriptional activation of various biological processes, including embryonic development, stem cell self-renewal, hematopoiesis, and tumorigenesis [79,80]. Elevated expression of ER alpha is frequently correlated with an increase in *ASH2L* expression in BC cell lines and primary BC [81]. In colorectal cancer, *ASH2L* acts positively by being recruited to the promoter region of genes associated with p53-mediated apoptosis, potentiating its function and inducing cell death [82]. These results provide promising clues for developing therapeutic targets in tumors related to ER alpha, such as breast cancer [80,83].

## 5. Conclusions

Concurrent germline SNVs and CNVs potentially associated with the risk of development of BC and CRC were identified in three HBOC/HBCC families. Prognostic markers and potential drug targets were also described. Early diagnosis of these patients and comprehensive genomic analysis are crucial to implementing more effective prevention or individualized treatment strategies that reduce the risk of a second primary tumor. Additional efforts are required to increase the number of BC/CRC families evaluated by the whole exome sequencing and clarify the impact of these alterations on cancer risk and prognosis.

## Figures and Tables

**Table 1:** Clinical characteristics of the three HBOC/HBCC patients included in this study.

Patient	Clinical Features	Relatives with Cancer
1	BC (64—left, T1N0M0, IDC-NE)	Sister (BC, 68)
	BC (64—right, TxN1M0, adenocarcinoma, NI)	
	CRC (71, TxNxMx, rectal carcinoma, NI)	
2	BC (55—left, T2N2aM0, IDC-NE—ER+/PR−/HER2−, surgery + CT + RT)	Sister (BC, 40 and CRC, 48)
	BC (55—right, T2N0M0, IDC-NE—ER+/PR+/HER2+, surgery + CT + RT)	Sister (thyroid cancer, 45)
	CRC (57, T3N2M0, TA, surgery + CT)	Maternal aunt (bone cancer, 52)
3	BC (48—right, TxN0M0, IDC-NE, surgery + CT + RT) CRC (56, T3N2M0, TA, surgery + CT)	Mother (bilateral BC, NI)
		Sister (BC, NI and pancreas cancer, NI)
		Maternal cousin (CRC, NI)
		Paternal cousin (BC, NI)
		Paternal cousin (BC, NI)

BC: breast cancer; CRC: colorectal cancer; CT: chemotherapy; ER: estrogen receptor; HBCC: hereditary breast and colorectal cancer syndrome according to [22,33]; HBOC: hereditary breast and ovarian cancer syndrome according to NCCN Guidelines Genetic/Familial High-Risk Assessment: Breast, Ovarian, and Pancreatic v2.2021; HER2: erb-b2 receptor tyrosine kinase 2; IDC-NE: invasive ductal carcinoma of non-specific type; NI: not informed; PR: progesterone receptor; RT: radiotherapy; TA: Tubular Adenocarcinoma; TNM staging system: T—tumor; N—lymph nodes; M—metastasis.

**Table 2:** Relevant single nucleotide variants and copy number variations identified in the three index patients.

**SINGLE NUCLEOTIDE VARIANTS**									
**Patient**	**Gene**	**cDNA**	**Protein**	**Type/Effect**	dbNSFP *	**GnomAD Variant Frequency**	**NCBI RefSeq**	**dbSNP 154 v2, NCBI**	**ACMG/ClinVar**
1	*CYP1B1*	c.1159G > A	p.Glu387Lys	Missense	4	0.00029	NM_000104.4	rs55989760	LP/LP
	*NTRK1*	c.16C > T	p.Arg6Trp	Missense	4	0.00325	NM_001012331.2	rs201472270	VUS/-
	*NFATC2*	c.1952G > A	p.Arg651His	Missense	4	0.00006	NM_173091.4	rs148347040	VUS/-
									(+47 VUS)
2	*SLC3A1*	c.1400T > C	p.Met467Thr	Missense	5	0.00250	NM_000341.4	rs121912691	VUS/LP
	*MAT1A*	c.826dupG	p.Ala276Glyfs*76	LoF	-	0.00001	NM_000429.3	rs763178849	LP/-
	*PTGIS*	c.824G > A	p.Arg275Gln	Missense	3	0.00098	NM_000961.4	rs61734270	VUS/LP
	*WRN*	c.95A > G	p.Lys32Arg	Missense	3	0.00305	NM_000553.6	rs34477820	VUS/-
									(+58 VUS)
3	*GNAQ*	c.286A > T	p.Thr96Ser	Missense	3	0.00440	NM_002072.5	rs753716491	VUS/-
	*PTCH1*	c.140G > T	p.Arg47Leu	Missense	2	0	NM_000264.5	rs775408408	VUS/-
									(+31 VUS)
**COPY NUMBER VARIATIONS**									
**Patient**	**Gene**	**Chrom**	**Start**	**End**	**Type**	**Other Genes**			**ACMG/ClinVar**
1	*PMS2*	7	5764955	6045662	Loss	*CCZ1, OCM, RNF216, RSPH10B*			VUS/VUS
	*POLE2*	14	50154854	50160008	Gain		*KLHDC1*		VUS/-
	*NOMO1*	16	14946287	14959012	Loss		-		VUS/-
									(+209 CNVs)
2	*PMS2*	7	6013030	6022622	Gain		-		VUS/-
									(+16 CNVs)
3	*PMS2*	7	6048628	6049129	Loss		*AIMP2*		VUS/P
	*ASH2L*	8	37914599	37963256	Loss		*EIF4EBP1*		VUS/-
	*POLE2*	14	50120873	50131392	Gain		-		VUS/-
									(+147 CNVs)

Chrom: chromosome; CNVs: copy number variations; LP: likely pathogenic; P: pathogenic; VUS: variant of uncertain significance. * Number of prediction algorithms (total of six) that indicated variants likely deleterious.

## Data Availability

The whole exome sequencing data will not be made publicly available due to restraints imposed by the ethics committee; requests for data can be made to the corresponding author.

## Supplementary Materials

The following supporting information can be downloaded at: https://www.mdpi.com/article/10.3390/genes14081580/s1. Table S1. List of germline variants (pathogenic, likely pathogenic and variants with uncertain significance) and copy number variations in three patients fulfilling HBOC criteria.
